# Recombinant human enamelin produced in *Escherichia coli* promotes mineralization in vitro

**DOI:** 10.1186/s12896-024-00875-0

**Published:** 2024-07-09

**Authors:** Monalissa Halablab, Lovisa Wallman, Johan Bonde

**Affiliations:** Division of Pure and Applied Biochemistry, Lund University, Lund, SE-221 00 Sweden

**Keywords:** Enamelin, Enamel, Enamel matrix protein, Expression, Purification, Optimization, Biomineralization

## Abstract

**Background:**

Enamelin is an enamel matrix protein that plays an essential role in the formation of enamel, the most mineralized tissue in the human body. Previous studies using animal models and proteins from natural sources point to a key role of enamelin in promoting mineralization events during enamel formation. However, natural sources of enamelin are scarce and with the current study we therefore aimed to establish a simple microbial production method for recombinant human enamelin to support its use as a mineralization agent.

**Results:**

In the study the 32 kDa fragment of human enamelin was successfully expressed in *Escherichia coli* and could be obtained using immobilized metal ion affinity chromatography purification (IMAC), dialysis, and lyophilization. This workflow resulted in a yield of approximately 10 mg enamelin per liter culture. Optimal conditions for IMAC purification were obtained using Ni^2+^ as the metal ion, and when including 30 mM imidazole during binding and washing steps. Furthermore, in vitro mineralization assays demonstrated that the recombinant enamelin could promote calcium phosphate mineralization at a concentration of 0.5 mg/ml.

**Conclusions:**

These findings address the scarcity of enamelin by facilitating its accessibility for further investigations into the mechanism of enamel formation and open new avenues for developing enamel-inspired mineralized biomaterials.

**Supplementary Information:**

The online version contains supplementary material available at 10.1186/s12896-024-00875-0.

## Background

Dental enamel is the hardest and most mineralized tissue in the human body. The unique mechanical properties of enamel come from a highly organized architecture of the hydroxyapatite crystals that compose about 95% of mature enamel. Despite being an almost completely inorganic tissue in the mature state, the nano- and microstructure of enamel is established in a protein rich extracellular enamel matrix early during enamel formation. Amelogenin is the most abundant protein of the developing enamel extracellular matrix and plays an essential role in the structural organization of the mineral phase in enamel [1]. A key feature of amelogenin is its ability to self-assemble into supramolecular aggregates and recently it has been demonstrated that amyloid-like fibrils of amelogenin termed nanoribbons are able to template organized formation of fibrous enamel-like hydroxyapatite crystals in vitro [2–4]. In addition, the observation of amyloid and fibrillar structures in vivo suggest that amelogenin nanoribbons are a key component in biological enamel formation [2, 3, 5, 6].

Despite the dominating prevalence of amelogenin in the developing enamel matrix, presence of non-amelogenin proteins is also critical for proper enamel formation. One key example is the protein enamelin that produces a severe phenotype with disrupted mineralization of the enamel matrix in a knockout mouse model [7]. Previous in vitro studies with the most abundant enamelin fragment, known as the 32 kDa fragment, isolated from pigs suggest that enamelin has a key role in promoting initiation/nucleation of calcium phosphate crystal growth which would explain the severe phenotype in the knockout mouse model [8–13]. In these studies, an increased activity of enamelin in presence of amelogenin has been observed, suggesting a co-operative effect between enamelin and amelogenin. A detailed understanding of the biomineralization events leading up to enamel and the interplay between the proteins involved is however still missing, calling for more research involving enamel matrix proteins.

In addition to their biological functions in biomineralization, the molecular components involved in enamel formation show promise as biocompatible agents. These components can be explored for biomimetic approaches, such as generating synthetic enamel-like materials [14], and for obtaining various mineralized materials. Such mineralized materials could be highly relevant for therapeutic or biomedical purposes like tissue engineering or regeneration of mineralized tissues like enamel, dentin, and bone. Obtaining enamel matrix proteins from natural sources is however cumbersome and pose ethical dilemmas, especially for the human proteins. Biotechnological production in a microbial system would thus be preferred and could make recombinant proteins available in a simple and cost-effective manner. Production of recombinant amelogenin in *Escherichia coli* has been previously reported and used extensively in the enamel research field [15, 16]. For recombinant enamelin, though previously explored as an in vitro Fam20C kinase substrate [17], there is no established production method to support its use as a mineralizing agent.

Therefore, in the present study we aim at producing the human 32 kDa fragment in an *Escherichia coli*-based expression system and evaluate its use as an agent for modulation of calcium phosphate mineralization in vitro. Herein we report of our methodology and the results from recombinant enamelin expression, purification, and subsequent in vitro mineralization assay.

## Methods

### Protein expression

An enamelin expression vector was constructed by cloning a DNA fragment encoding the 32 kDa fragment of human enamelin (amino acid 173–280), including an N-terminal His_6_-tag and enterokinase site, between the *Nde*I and *Bam*HI sites in the pET11a vector (Novagen) (Supplementary information: Figure S1, Figure S2). The enamelin expression vector was verified by Sanger sequencing and subsequently transformed into *E. coli* BL21 (DE3) cells for protein expression. Cells were inoculated in LB-medium containing 100 μg/ml ampicillin and were grown overnight in glass tubes at 37 °C with shaking at 150 rpm. Optical density at 600 nm (OD_600_) of the overnight culture was measured and the culture was diluted to a final OD_600_ = 0.015 in TB-medium (tryptone 12 g/l, yeast extract 24 g/l, glycerol 4 ml/l, 17 mM KH_2_PO_4_, 72 mM K_2_HPO_4_) containing ampicillin (100 μg/ml). The cultivations were carried out in shake flasks at 170 rpm and 37 °C, and protein expression was evaluated at various conditions. Protein expression was induced with 0.2, 0.5 or 1 mM IPTG when cultures reached OD_600_ ∼ 1.5 (4 h post-inoculum), and the cells were then cultured for an additional 4–20 h post-induction. Cells were harvested by centrifugation (4 500 g for 10 min at 4 °C), re-suspended in binding buffer (50 mM NaH_2_PO_4_, 300 mM NaCl, pH 7.4) to 20% of the culture volume, and then lysed by sonication. The supernatant and lysate pellet were separated by centrifugation at 20 000 g for 20 min at 4 °C.

### Protein purification

The supernatant (raw extract) with or without imidazole (0–50 mM) was mixed with 200 μl IMAC Sepharose™ 6 Fast Flow media (GE healthcare) for small-scale batch purifications and optimizations. Briefly, for small-scale purification, four metal ions were examined: Ni^2+^, Cu^2+^, Co^2+^, and Zn^2+^, and they were immobilized according to the manufacturer’s instruction. The extract was mixed with the media for 20 min and then the media was separated from the unbound fraction with a brief centrifugation. The media was then washed with 2 × 1 ml binding buffer with or without imidazole (0–50 mM) to remove non-specifically bound proteins and the remaining protein was eluted from the gel with 200 μl of elution buffer (50 mM NaH_2_PO_4_, 300 mM NaCl, 500 mM imidazole, pH 7.4).

For column-based purification the raw extract (containing 0–30 mM imidazole) was loaded onto a Ni^2+^-charged 5 ml GoBio™ Mini IDA column (Bio-works) and then washed with 3 column volumes (CV) binding buffer with or without imidazole (0–30 mM). The elution phase started with a 3 CV linear gradient elusion to 100% elution buffer, followed by a subsequent 10 CV isocratic elution at 100% elution buffer. The flowrate during the purification was kept at 2 ml/minute.

The purification was recorded by the chromatography system (ÄKTA Avant, GE Healthcare) by following absorbance at 280 nm. Elution fractions were collected, dialyzed against ddH_2_O and lyophilized. For subsequent experiments, the lyophilized enamelin weighed on a bench-top balance was dissolved in water to make a 10 mg/ml stock solution.

### Enterokinase digestion

To cleave the His_6_-tag from the protein, recombinant enterokinase fused with a His_6_-tag (0.3 mg/ml), as supplied by SinoBiological, was added to enamelin. The digestion reaction was performed in a final volume of 100 μl in water at 25 °C with 0.1 mg/ml enamelin and 0.03 mg/ml enterokinase.

### SDS-PAGE

SDS-PAGE was performed using pre-cast 4–20% gel according to manufacturer’s description (Bio-Rad). The samples to be analyzed were mixed with equal volume of 2× SDS-loading buffer (62.5 mM Tris-HCl, pH 6.8, 2% (w/v) SDS, 10% (v/v) glycerol, 5% (v/v) β-mercaptoethanol, and 0.0125% (w/v) bromophenol blue) and heated at 95 °C for 10 min before loaded on the gel. Some samples of purified enamelin were pre-treated with urea or guanidine-HCl prior to SDS-PAGE to test dissociation of potential multimers (see details in Supplementary Information, Figure S4). Sampling during the cultivations was carried out as previously described [18] such that an equal amount of lysed cells was in each sample making it is possible to compare the enamelin levels. SDS-PAGE gel was stained with Coomassie blue and photographed with EPI White illumination using Gel-doc XR (Bio-Rad).

### Mass-spectrometry

Mass spectrometry (MS) was carried out using in-gel digestion to confirm the identity of enamelin. The protein band was excised from the SDS-PAGE gel, washed, dehydrated, and digested with trypsin as previously described [19]. 1 μl peptide sample was mixed with 0.5 μl matrix solution, consisting of 5 mg/ml α-cyano-4-hydroxy cinnamic acid, 80% acetonitrile, 0.1% TFA, and added to a MALDI stainless steel plate. MS and MS-MS spectra were acquired using an Autoflex Speed MALDI TOF/TOF mass spectrometer (Bruker Daltonics, Bremen, Germany) in positive reflector mode. The Swissprot database of all entities, allowing one missed cleavage and methionine oxidation, as well as a custom database with the theoretical enamelin sequence was used for comparison with the experimental masses. Identification of proteins were carried out with the Mascot Daemon software (version 2.4, Matrix Science). In addition to analyzing the trypsin digested protein, MALDI TOF MS operated in linear mode was used to analyze the intact protein.

### In vitro biomineralization assay

The ability of recombinant enamelin to promote mineralization in calcium phosphate solutions was investigated using turbidity-based assays, similar to how to mineralization has been assessed previously [20]. The mineralization buffer (CaP) contained 21 mM KH_2_PO_4_, 34 mM CaCl_2_ and 100 mM acetic acid (HAc) adjusted to pH 5.5 with KOH. The control buffer (P) was the same as the mineralization buffer but without CaCl_2_. Enamelin was added to a final concentration of 0.5 mg/ml from a 10 mg/ml stock solution in water. Samples were prepared in triplicates in wells of a sterile 96-well plate and incubated at 37 °C. OD_600_ measurements were performed using SPECTROstar^Nano^ plate reader (BMG Labtech) at different time points during the experiment. To verify the nature of the precipitate formed in the mineralization assay the soluble and insoluble (precipitate) fractions were analyzed with SDS-PAGE and Fourier Transform Infrared spectroscopy (FTIR). This included control samples with mineralization buffers lacking one of the two mineral components (CaCl_2_ and KH_2_PO_4_, respectively). Samples were taken directly from the mineralization reaction for SDS-PAGE analysis (Total). The reactions were then centrifuged to pellet the precipitates. Samples were taken from the supernatants for SDS-PAGE analysis (Sup). Pellets were washed with ddH_2_O and samples were taken for SDS-PAGE (Pellet). Pellets were then air dried and analyzed with FTIR on a NICOLET iS5 using an iD5 ATR-Diamond.

## Results and discussion

### Protein expression

**Fig. 1:**
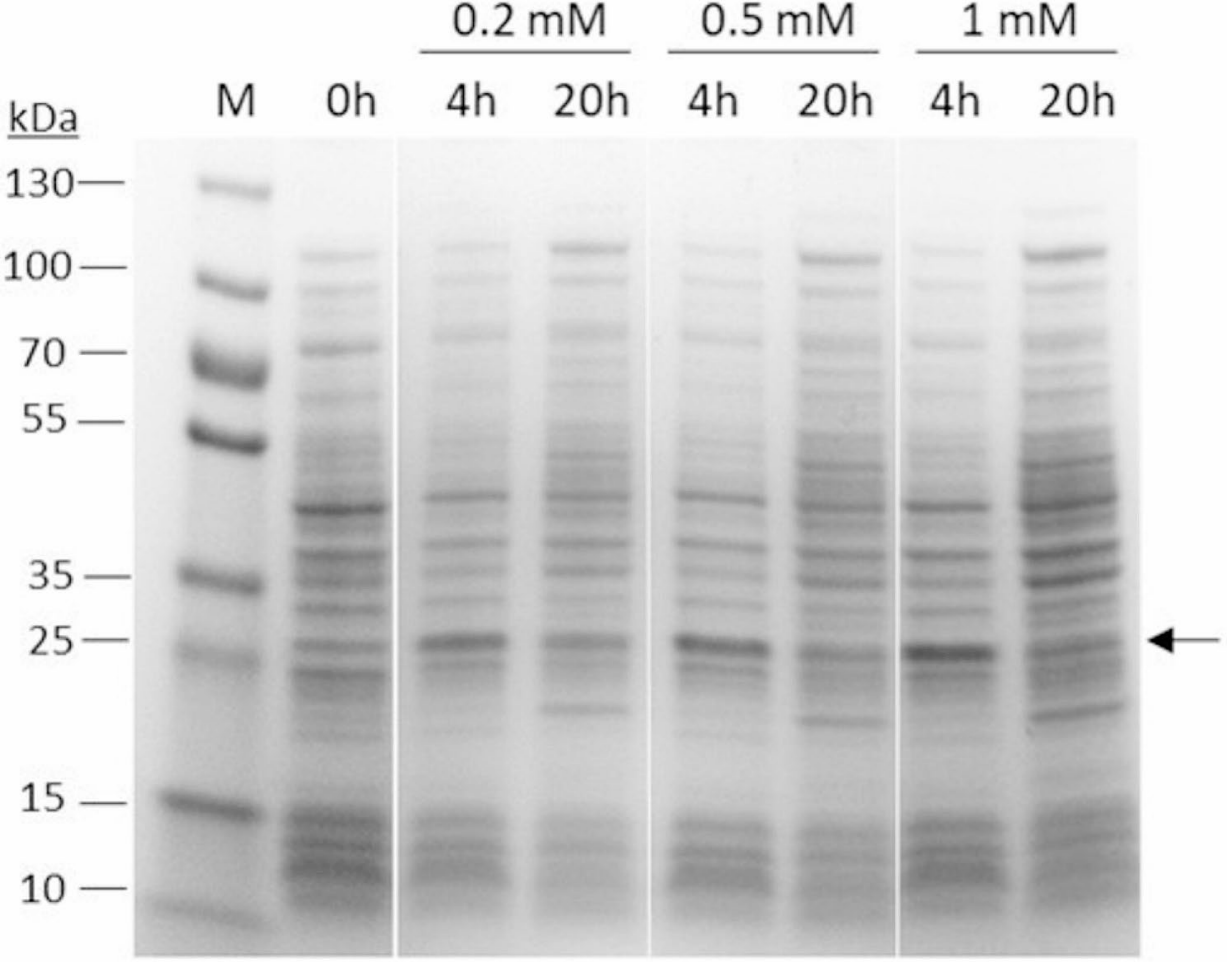
SDS-PAGE analysis of enamelin levels at different times after induction with various IPTG concentrations.*E. coli* BL21 (DE3) cells carrying the enamelin expression vector were grown and induced with 0.2-1 mM IPTG for 4–20 h. Samples were normalized to allow comparison of the enamelin levels. The arrow indicates the proposed enamelin band

For recombinant production of human enamelin in *E. coli*, we used transformed *E. coli* BL21 (DE3) cells carrying the enamelin expression vector. We grew the cells in TB-medium and induced protein expression with varying IPTG concentrations (0.2-1 mM) for 4 and 20 h. OD_600_ after 4 h induction was typically in the range 3–5, and after a total of 20 h induction the OD_600_ reached 12–15. At all IPTG concentrations tested, 4 h induction resulted in the highest level of enamelin expression as analyzed by SDS-PAGE (Fig. 1). The enamelin levels after 20 h induction were, however, significantly lower, despite the higher final OD_600_. This suggests that enamelin expression pose a stress on the cells that cause enamelin levels to drop as the cells continue to grow. As for the IPTG concentration, 1 mM resulted in the highest expression level (4 h induction), suggesting that the best approach for enamelin expression is a high inducer concentration in combination with a relatively short induction phase.

**Fig. 2:**
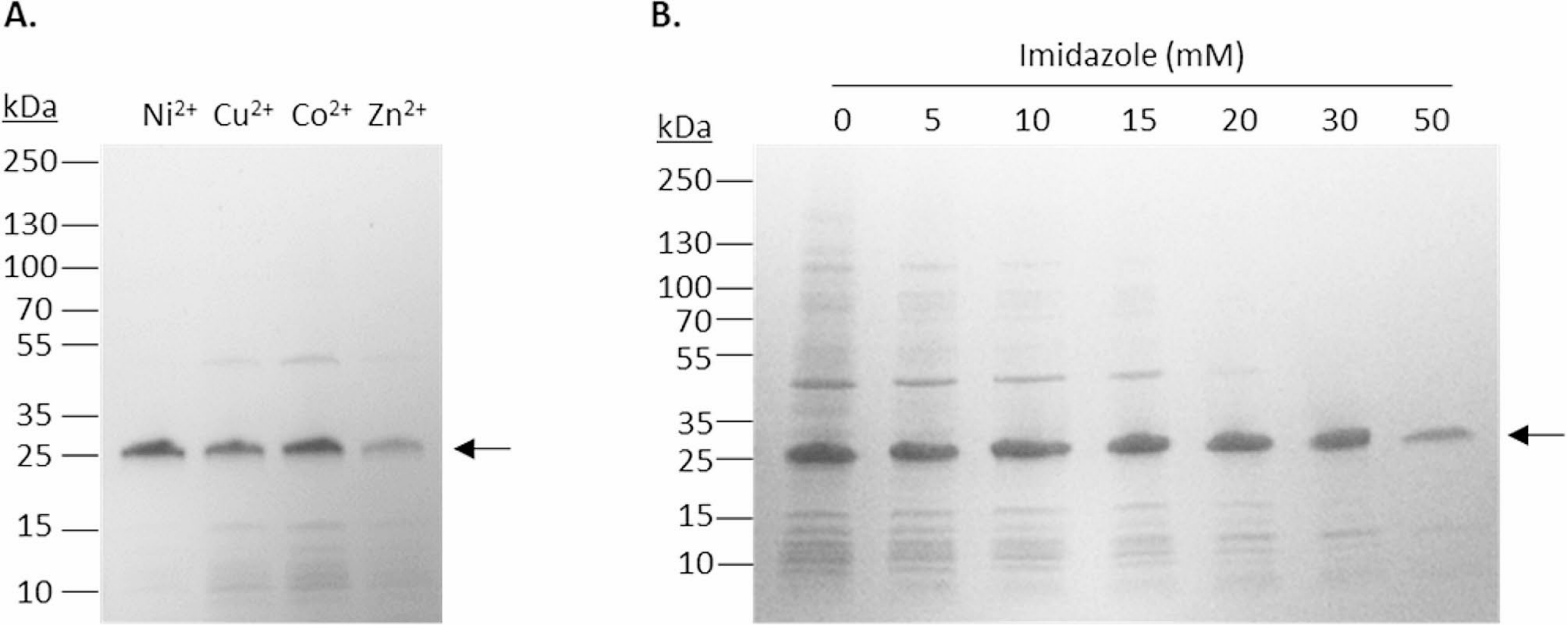
SDS-PAGE analysis of batch-mode enamelin purification. (A) Purification of enamelin was carried out using four different metal ions (Ni^2+^, Cu^2+^, Co^2+^, Zn^2+^) with an imidazole concentration of 30 mM during binding and washing. The image shows elution fractions from using the metal ions indicated above each corresponding well. (**B**) Elution fractions from Ni^2+^-based purifications of enamelin using different imidazole concentrations (0–50 mM) during binding and washing. The concentration is indicated above each corresponding well

The most abundant SDS-PAGE band appearing after induction of enamelin expression (and absent in the uninduced control) had an apparent molecular mass of ∼25 kDa, based on the migration in the gel. This is significantly higher than the theoretical value (13.3 kDa) expected from the amino acid sequence. The SDS-PAGE sample treatment and conditions used are highly denaturating and should disrupt protein-protein interactions that otherwise could lead to multimeric forms and higher apparent molecular weight. Although multimeric forms during SDS-PAGE cannot be completely ruled out, another explanation could be an abnormal electrophoretic migration of enamelin compared to the proteins in the molecular weight standard. This could be because of reduced decoration with SDS, or because of other structural features affecting the interaction with the polyacrylamide network. A similar abnormal electrophoretic migration, although not as pronounced, has been observed previously also for amelogenin [16, 21] and is common for proteins having disordered regions [22]. The identity of the 25 kDa band is corroborated by its accumulation during purification (Fig. 2) and the subsequent MALDI-TOF mass fingerprinting analysis, which identified it as enamelin.

In the MS analysis, tryptic peptides corresponding to the N-terminal region of the recombinant enamelin could be identified. Observed monoisotopic masses included 2 537.248 Da (residue 1–20) and 995.538 Da (residue 13–20), and sequence information obtained via MS-MS were in line with the expected sequence of recombinant enamelin (Supplementary information: Figure S3 and Table S1). Linear mode MS analysis of the purified intact protein generated peaks that corresponded well with the monomeric theoretical molecular mass of our recombinant enamelin (13 261 Da) for instance a 1 H + ion at m/z 13 259 as well as its corresponding 2 H + ion (see spectra and peak assignment in Supplementary information: Figure S5). A minor peak (1 H+) corresponds to the molecular mass of dimeric enamelin, which could indicate some presence of dimers in the sample or be a result of dimerization during the MS analysis. However, if stable dimers were abundant in the initial sample a stronger signal for the dimer peak would have been expected. The intact protein MS data thus suggest that our recombinant protein migrating at around 25 kDa in SDS-PAGE has a molecular weight of 13 kDa, consistent with a monomer. To further assess potential dimerization, we tested if pre-treatment with urea or guanidine-HCl had an impact on the molecular mass observed in SDS-PAGE. Even with the pre-treatment, the band at 25 kDa was still observed on the gel (Supplementary information: Figure S4), meaning that potential dimers would have to be stable at highly denaturating conditions, which we consider less probable. Taken together, the data suggests that enamelin likely has an abnormal electrophoretic mobility in SDS-PAGE which makes it appears as a 25 kDa band instead of the expected 13 kDa band. Interestingly, in a previous study [23] with the native porcine 32 kDa enamelin fragment glycosylations were removed with glycopeptidase F, which lowered the apparent molecular mass in SDS-PAGE analysis from 32 kDa to approximately 23–25 kDa, indicating a similar behavior to our recombinant protein.

### Protein purification

Since the protein was engineered with a His_6_-tag at its N-terminus, we used immobilized metal ion affinity chromatography (IMAC) to promote a simple purification. The effect of imidazole concentration and choice of metal ion were tested in batch-mode to determine optimal purification conditions, and by analyzing the eluted fractions on an SDS-PAGE gel we could determine that Ni^2+^ metal ion (Fig. 2A) and the 30 mM imidazole condition (Fig. 2B) resulted in the highest yield and purity of enamelin. Although Ni^2+^ was the optimal metal ion for enamelin purification, other metal ions were also binding enamelin. Co^2+^ bound enamelin to a similar degree as Ni^2+^ but was also binding several other proteins based on SDS-PAGE (Fig. 2A). Cu^2+^ and Zn^2+^ bound enamelin to a lesser degree and we thus found the order of preference for metal ion to be Ni^2+^ > Co^2+^ > Cu^2+^ >> Zn^2+^. Ni^2+^ was therefore used for the column-based purification. Besides the choice of metal ion, presence of imidazole during binding and washing also had large impact on the level of contaminating proteins in the eluate (Fig. 2B). Up to 30 mM imidazole had a positive effect and reduced the amount of protein contaminants without significantly impacting enamelin binding. At 50 mM imidiazole the level of enamelin binding was significantly reduced, and we therefore deem this concentration as too high.

**Fig. 3:**
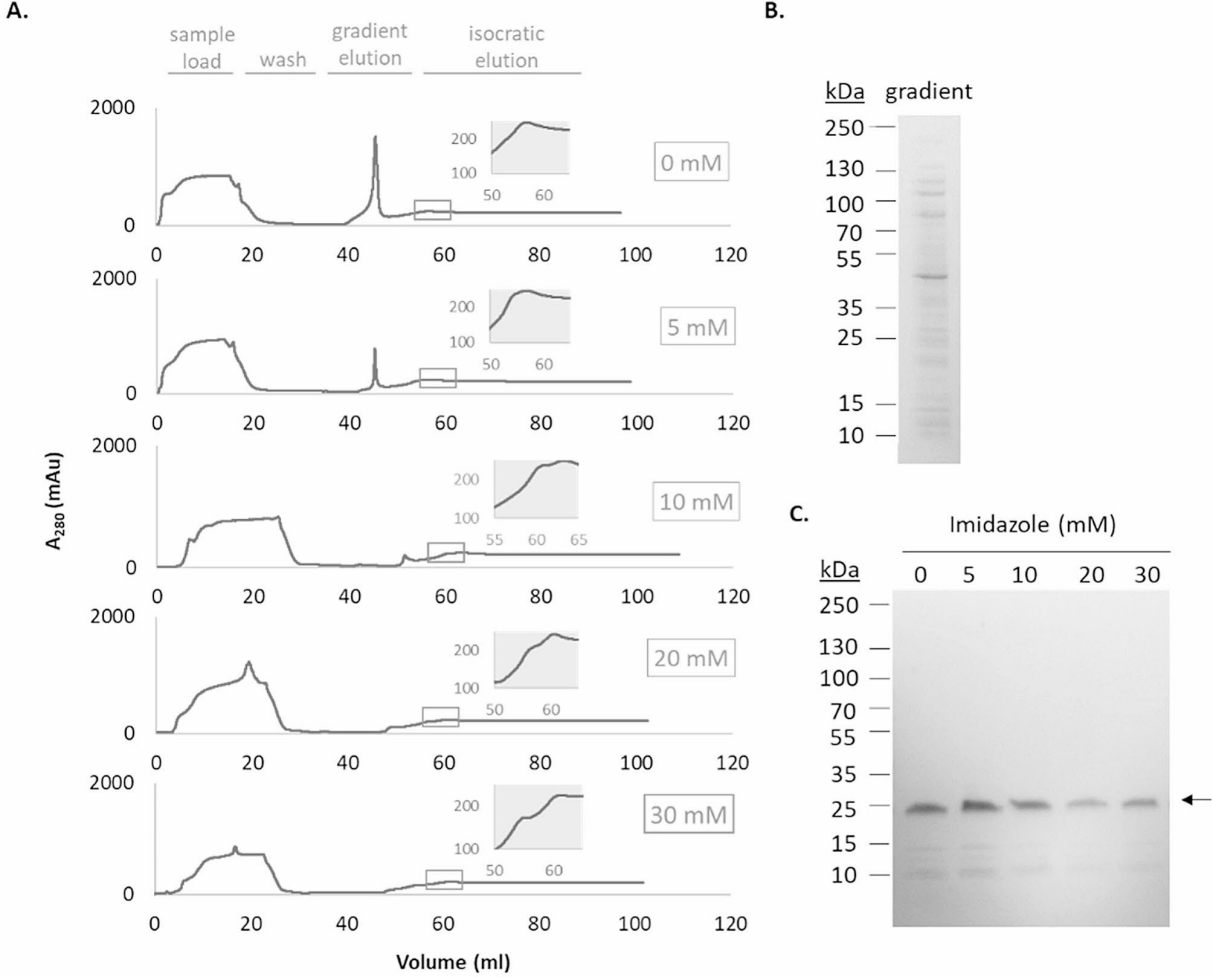
Column-based purification of enamelin. (**A**) Chromatograms showing purification of enamelin using different imidazole concentrations (0–30 mM) during loading and washing steps. Main elution peaks are shown (grey box). (**B**) SDS-PAGE analysis of the gradient peak fraction from 0 mM experiment. (**C**) Elution fractions from the column purifications analyzed with SDS-PAGE. The arrow indicates the enamelin band. Imidazole concentrations during binding and washing are indicated above each corresponding well

We then scaled up the purification of enamelin by using a column-based set-up, and different concentrations of imidazole during the binding and washing steps were tested to determine optimal purification conditions. Based on the chromatograms (Fig. 3A) the imidazole concentration had a big effect on the gradient part of the purification and which proteins that eluted during this step. A major peak is observed during the gradient step at the lower imidazole concentrations but decreases in response to increasing imidazole concentrations. Based on SDS-PAGE (Fig. 3B) this peak is composed of a range of non-target proteins and is not enriched in enamelin. At all conditions tested enamelin was eluting during the isocratic phase of the elution. Though the enamelin peak is somewhat obscured by the absorbance of imidazole the elution peak appears rather broad, suggesting some interaction between enamelin and the chromatography media even at the highest imidazole concentration (500 mM). Interestingly, in the column-mode purification the effect of imidazole on the purity of the eluted enamelin was much less pronounced than in the batch-mode purification (Fig. 3C). Though the difference in chromatography media may have contributed to some degree, the major reason for the difference is likely due to the more efficient separation obtained in column-mode by using a combination of gradient and isocratic elution, which removed weakly bound proteins to larger degree. However, binding of non-target proteins at low imidazole concentrations will have a negative effect on the column capacity and such conditions should thus be avoided. Based on the different conditions tested, we deemed the best imidazole concentration to use in binding and washing to be 30 mM, considering the purify of the eluted protein and the reduced binding of non-target protein, albeit with some enamelin loss compared to lower imidazole concentrations. After the final dialysis and lyophilization approximately 10 mg enamelin per liter culture could be obtained with 90% purity.

Other studies have reported use of recombinant enamelin, although not for mineralization purposes like in the present study [17, 24]. It is hard to compare the recombinant proteins from the different studies, and their production, since the previous studies were not focusing on those aspects.

### Enterokinase digestion

**Fig. 4:**
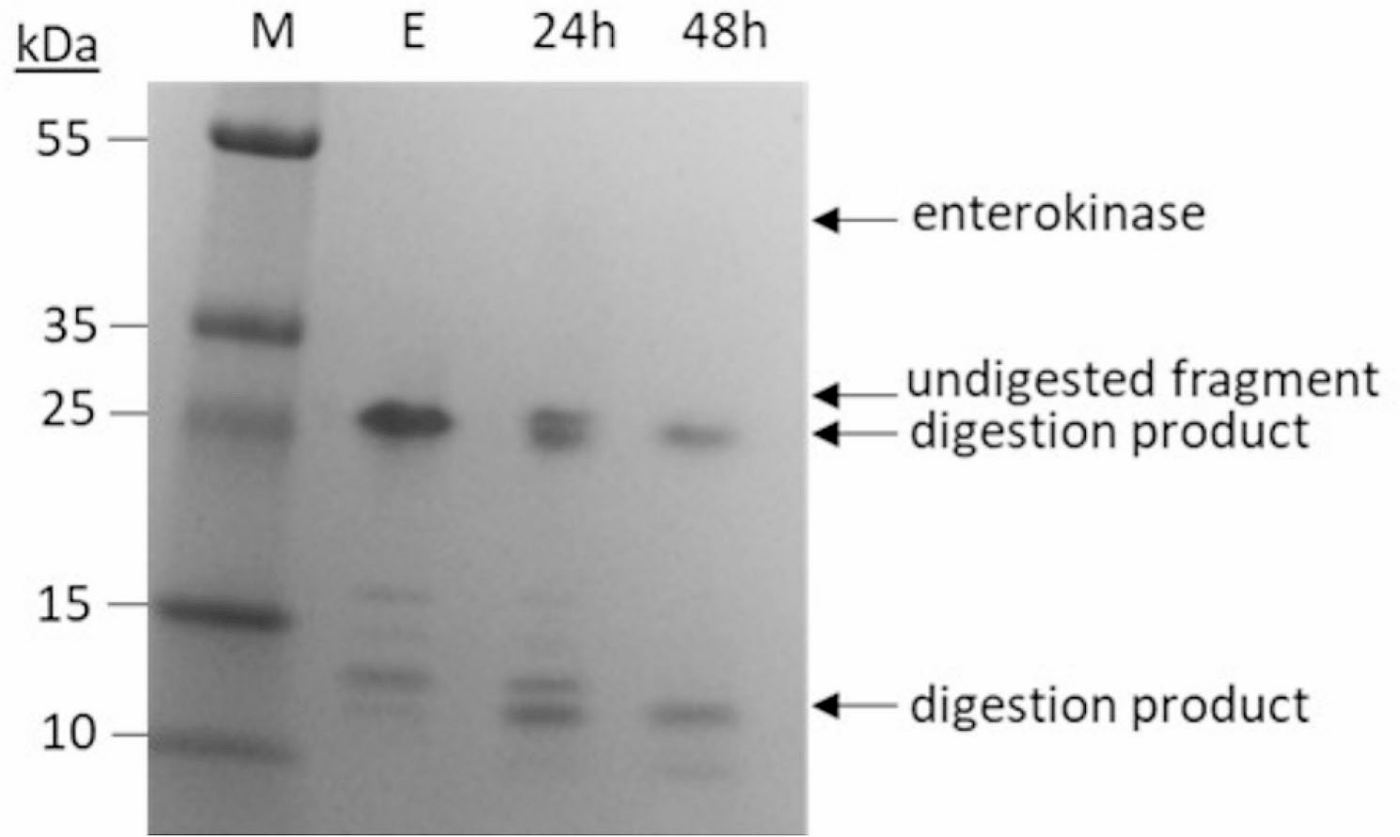
Enterokinase digestion. Enamelin (E) was digested with enterokinase in water at 25 °C and digestion samples were analyzed by SDS-PAGE as shown. The undigested protein and the digestion products appearing upon incubation (24 h and 48 h) with enterokinase are indicated with arrows

In the recombinant protein there is an enterokinase cleavage site immediately downstream of the His_6_-tag to facilitate proteolytic removal of the His_6_-tag from enamelin. Digestion could be observed after SDS-PAGE as seen by the lower molecular weight bands corresponding to digestion fragments (Fig. 4). However, after evaluating a range of digestion conditions, a clear cleavage pattern could only be observed at very high relative amounts of enterokinase, corresponding to a 3.3:1 ratio (by weight) between enamelin and enterokinase. The high amount of enterokinase needed could indicate that the recognition site was not readily accessible for the enzyme, and this made the tag removal process ineffective and costly. The disappearance of the untagged 25 kDa band after 48 h digestion suggest that all the tagged protein was processed by the enzyme, however, due to the poor efficiency of the reaction the cleavage products were not further characterized, and the tagged protein was used for the rest of the study. Potential effects of the tag on the properties of the protein were thus not investigated.

### Activity of recombinant enamelin

**Fig. 5:**
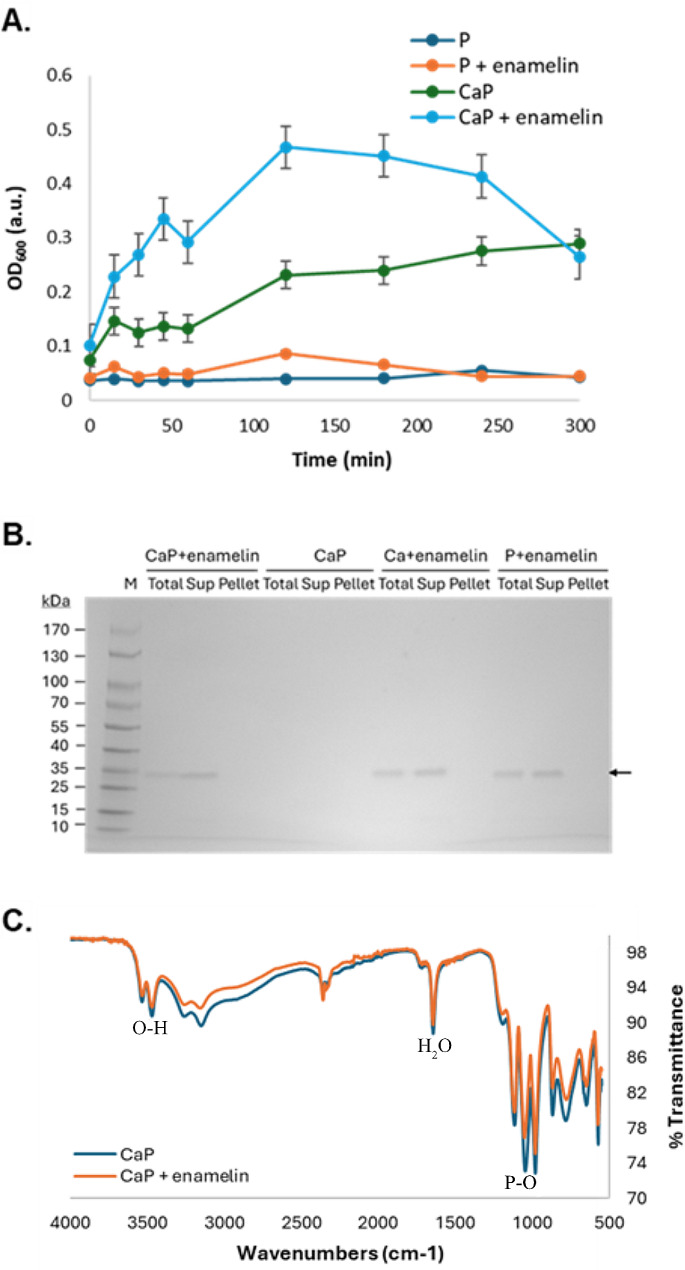
In vitro mineralization activity of recombinant enamelin. (**A**) The curves show turbidity over time as a measure for calcium phosphate precipitation. Mineralization buffer containing enamelin at 0.5 mg/ml (CaP + enamelin) is compared to buffer alone (CaP). Non-mineralizing buffer without (P) and with enamelin (*P* + enamelin) were used as negative controls. Three separate experiments were carried out, and the values plotted are the mean values and the error bars indicate the standard error of the mean. (**B**) SDS-PAGE analysis of samples taken during mineralization experiment with enamelin in combination with calcium- and phosphate ions (Ca, P, CaP). Soluble (sup) and insoluble (pellet) samples are taken after centrifugation of the complete sample (total). The enamelin band is not present in any of the pellet samples, suggesting that the protein is soluble during the mineralization assay. (**C**) FTIR spectra of the CaP mineralization sample with and without enamelin. Bands expected in calcium phosphate mineral can be observed and associated to OH (3533-3469-3255–3151 cm^− 1^), PO (1116-1046-980 cm^− 1^) and mineral bound H_2_O (1644 cm^− 1^), indicating presence of brushite [25–27]

The effect of enamelin on calcium phosphate mineralization was examined in vitro to assess the activity and possible application of the recombinant protein. The assay conditions used are supersaturated with respect to calcium phosphate, which means over time precipitation of solid calcium phosphate will occur causing an increase in sample turbidity that can be measured. In our control sample without enamelin (CaP) this manifested as a slow increase in turbidity over time (Fig. 5A). When enamelin is present (CaP + enamelin), the increase in turbidity was significantly accelerated suggesting that mineralization is promoted by the presence of the protein. At the later time points the OD_600_ values appeared to diminish, concomitant with formation of larger precipitates, and the dropping curve is likely due to distorted turbidity readings related to particle size, rather than reduced calcium phosphate precipitation. Enamelin solutions at non-mineralizing conditions (absence of CaCl_2_), however, displayed no significant turbidity during our assay (*P* + enamelin), suggesting that the protein itself does not disturb the measurement.

The mineralization reactions were also analyzed by SDS-PAGE and FTIR. SDS-PAGE analysis of samples taken during mineralization showed presence of the enamelin band in the supernatant samples indicating that it was soluble in the mineralization buffers (Fig. 5B), and the absence of the enamelin band in the Pellet samples suggest that the pelleted precipitates do not contain protein (Fig. 5B). Furthermore, the FTIR spectra resulting from analysis of the precipitates from the mineralization experiments showed absorbance bands corresponding to P-O, O-H, and mineral bound H_2_O that can be expected from calcium phosphate mineral (Fig. 5C). Based on comparison with previous studies our FTIR spectra mostly resembles that of brushite, suggesting this can be the main component at the assay conditions [25–27]. However, we don’t exclude that other calcium phosphate phases also can be present. Samples with (CaP + enamelin) and without (CaP) both showed similar spectra, suggesting that our recombinant enamelin did not significantly change the composition of the mineral phase. For the control mineralization experiments in buffers lacking CaCl_2_ and KH_2_PO_4_, respectively, no precipitates formed and could thus not be analyzed by FTIR.

In our study, the mineralization promoting behavior of enamelin was consistent at an enamelin concentration of 0.5 mg/ml, however, at lower concentrations (20–100 μg/ml) the effect on mineralization was not always coherent in relation to the enamelin concentration (Supplementary information: Figure S6) suggesting a non-linear dose response. A previous study on porcine enamelin co-assembled with amelogenin reported a nonmonotonic mineral nucleation behaviour in response to different enamelin concentrations [8], and it is possible that our recombinant enamelin share similar properties at low concentrations. The enamelin concentration that reliably promoted mineralization in our study (0.5 mg/ml) was significantly higher than the concentrations of porcine enamelin used in previous studies, which typically were in the 1–40 μg/ml range [8, 11–13]. During enamel formation (secretory stage) the total protein concentration in the extracellular matrix can reach up to 300 mg/ml [28] out of which enamelin comprise 1–5%, suggesting that biological enamelin concentrations likely exceed the ones tested also in the present study.

The *E. coli* expression system used for enamelin is not able to perform post-translational modifications like glycosylations and phosphorylations known to be present on the native protein [23, 29]. Mutation at a phosphorylation site within the 32 kDa enamelin fragment (Ser 216) has been previously reported to cause the genetic enamel disorder *Amelogenesis imperfecta* [30], suggesting that enamelin phosphorylation is important for the biological enamel formation process. An additional phosphorylation site, outside the 32 kDa fragment (Ser 55), has also been seen to affect enamel formation in mouse models [31]. Although the phosphorylation of enamelin appears important for proper enamel formation based on the mutated proteins, the mechanistic explanations for this are still unclear and could be due to effects not directly involving mineralization, like regulation of cellular events.

Importantly, despite missing post-translational modifications the recombinant enamelin in the present study still demonstrate mineralization promotive properties in vitro that could be relevant to biological enamel formation. It should be mentioned that the missing post-translational modifications may still impact the activity of our recombinant protein compared to the native enamelin present in the enamel matrix. The amino acid sequence of the 32 kDa fragment used in our study has a region dense in negatively charged amino acids (Supplementary information: Figure S1), primarily glutamic acid, which potentially could interact with mineral ions and lead to the observed effect on mineralization. Polyanionic proteins such as bone sialoprotein, osteopontin, or dentin phophophoryn, are critical for the formation of other mineralized biological materials like bone and dentin and are involved in mineralization events like nucleation or formation of amorphous mineral precursors [32–34]. In these processes, an organic scaffold, typically composed of collagen fibrils, is involved in directing the mineral crystallization in concert with the polyanionic proteins and templates the proper morphology and architecture of the mineralized material. For the enamel system, which is lacking collagen, a similar process has been observed in vitro using self-assembled amelogenin nanoribbons in combination with poly-aspartic acid [2]. During biological enamel formation, however, enamelin could have the mineral interacting role taken by poly-aspartic acid in the in vitro system [2, 5]. How such events occur during enamel formation still needs to be further investigated and use of recombinant enamelin fragments, like the one we produced in the present study, can then be highly useful.

Though the effect of recombinant enamelin on mineralization events can be further investigated to better understand mechanistic details, the present study demonstrates the potential of biotechnologically produced components from the enamel formation machinery as bioactive agents for modulating mineralization in vitro. Recombinant enamelin could thus be promising to explore for future biomaterial applications related to mineralized tissues.

## Conclusion

Recombinant human enamelin (32 kDa fragment) could be successfully expressed in *E. coli* and purified via an easy purification procedure based on immobilized metal ion affinity chromatography. Despite lacking the posttranslational modifications present on the native protein, the recombinant enamelin demonstrated ability to modulate mineralization in supersaturated solutions by promoting calcium phosphate precipitation. This implies that recombinant enamelin could be utilized for in vitro investigations of mineralization processes relevant for enamel formation or explored as a mineralization inducing agent for biomaterial applications.

### Electronic supplementary material

Below is the link to the electronic supplementary material.

Supplementary Material 1

Supplementary Material 2

## Data Availability

The data and materials that support the findings of this study are available from the corresponding author upon reasonable request.
