# Supplementary material for: Recombinant human enamelin produced in Escherichia coli promotes mineralization in vitro
Source: BMC Biotechnol. 2024 Jul 9;24:48. doi: 10.1186/s12896-024-00875-0 (PMC11234762; doi:10.1186/s12896-024-00875-0)
Supplement: Supplementary file 1 — Supplementary Material 1 [file 12896_2024_875_MOESM1_ESM.pdf]

Recombinant human enamelin produced in *Escherichia coli* promotes mineralization *in vitro*.

Monalissa Halablab[1], Lovisa Wallman[1] and Johan Bonde[1*]

Affiliations:
[1]Division of Pure and Applied Biochemistry, Lund University, Lund, SE-221 00, Sweden
[*]Corresponding author email: johan.bonde@tbiokem.lth.se (J.B.)

SUPPLEMENTARY INFORMATION

Content:

Figures S1-S6
Table S1

MHHHHHHDDD DKAPWQIPQR LPPPGYGRPP ISN**EE**GGNPY FGYFGYHGFG
GRPPYYS**EE**M F**E**Q**D**F**E**KPK**E** **ED**PPKA**E**SPG T**E**PTANSTVT **E**TNSTQPNPK
GSQGGN**D**TSP TGNSTPGLNT G

Figure S1. Amino acid sequence of human 32 kDa enamelin used in the study. The enamelin amino acids (highlighted in grey) correspond to residue 173 to 280 in full length enamelin (Uniprot accession number Q9NRM1). The negatively charged residues in enamelin are indicated in bold. The His$_6$-tag (magenta) and enterokinase site (yellow) are also highlighted in the sequence. Potential trypsin cleavage sites (after K and R residues) are indicated with a triangle (▼).

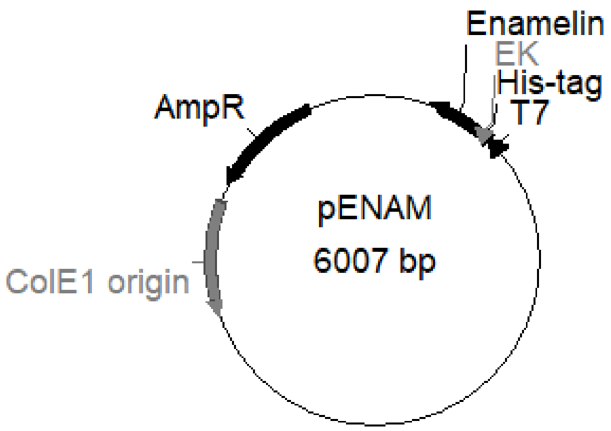

Figure S2. Plasmid map. Plasmid pENAM is a derivative of pET11a (Novagen) and expresses enamelin173-280. The enamelin gene fragment in pENAM belongs to human enamelin (amino acid residues 173 to 280 in Uniprot record Q9NRM1) and is codon optimized for *E. coli*. The protein is tagged with a His-tag and there is an enterokinase site (EK) in-between for His-tag removal. Theoretical molecular weight of the encoded protein is 13 261 Da.

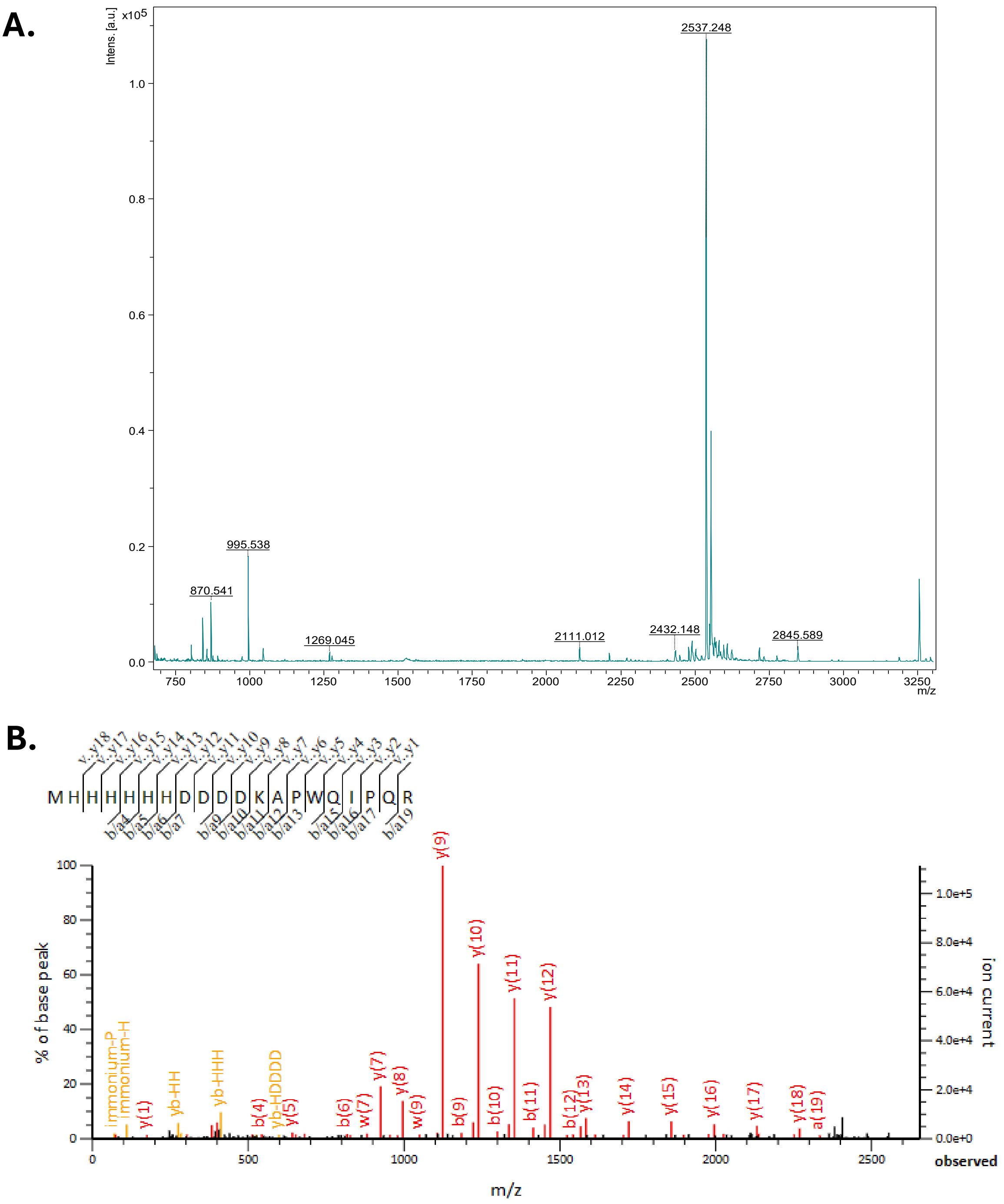

Figure S3. Mass spectroscopy identification of the 25 kDa band from SDS-PAGE. Panel **A:** MALDI-TOF MS spectrum of the peptides obtained after trypsin digestion of the 25 kDa band. The MS peak at m/z 2537.248 Da corresponds to amino acids 1-20 of the recombinant protein (MHHHHHHDDDDKAPWQIPQR). Panel **B**: MS-MS spectrum obtained by fragmentation of the 2537.248 Da peptide. The fragmentation pattern in relation to the amino acid sequence is indicated at the top left, together with the related masses. The identified masses are given in Table S1.

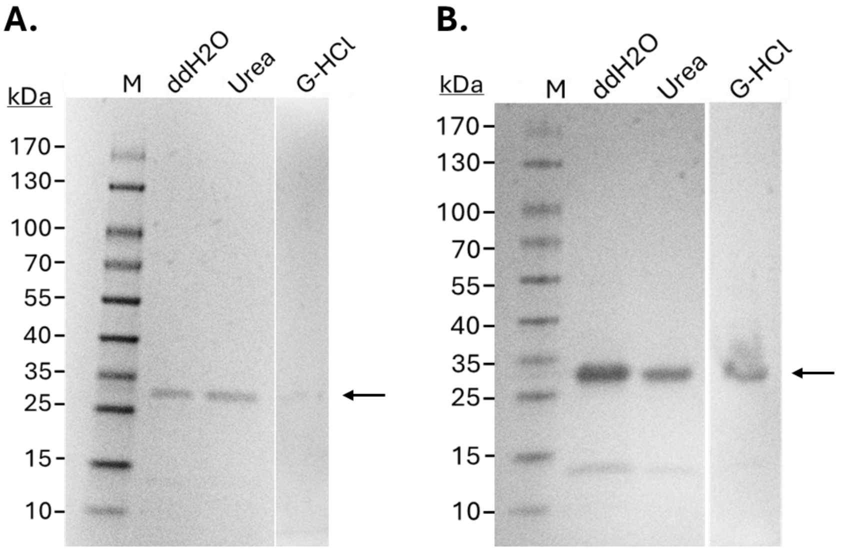

Figure S4. SDS-PAGE analysis of Urea and Guanidine-HCl denaturation. A. 100 µl enamelin was mixed with 900 µl 8 M Urea, 6 M Guanidine HCl or ddH2O, left at ambient temperature for 15 min and analyzed by SDS-PAGE. **B.** 10 µl enamelin was mixed with 10 µl 8 M Urea, 6 M Guanidine HCl or ddH2O, left at ambient temperature for 15 min and analyzed by SDS-PAGE**.**

A.

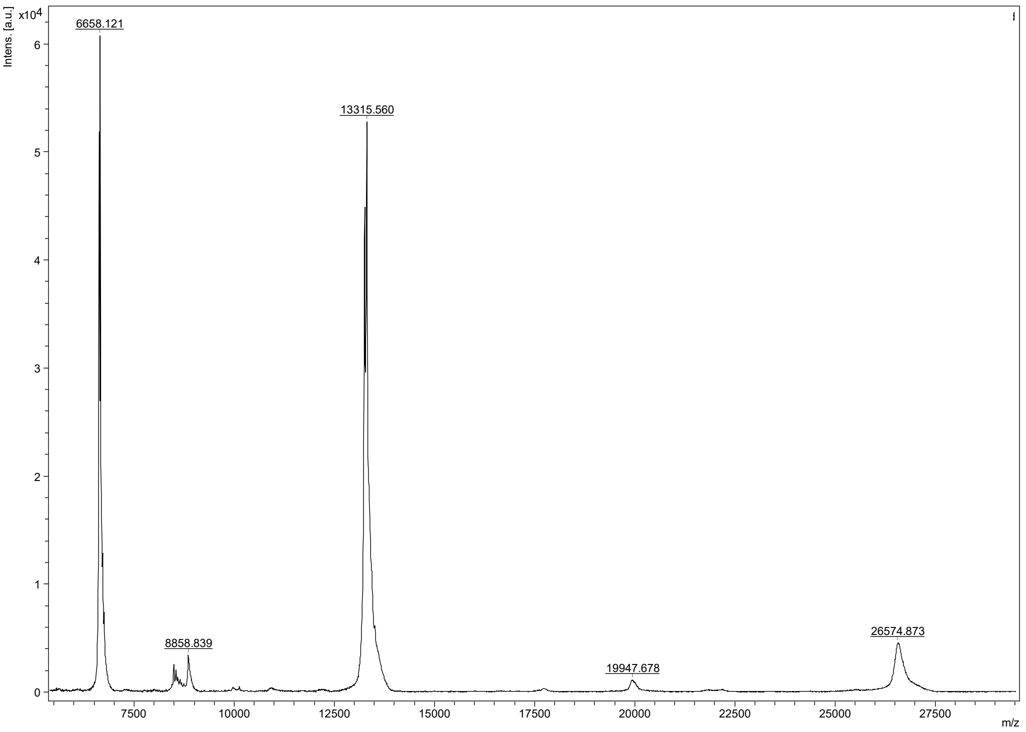

B.

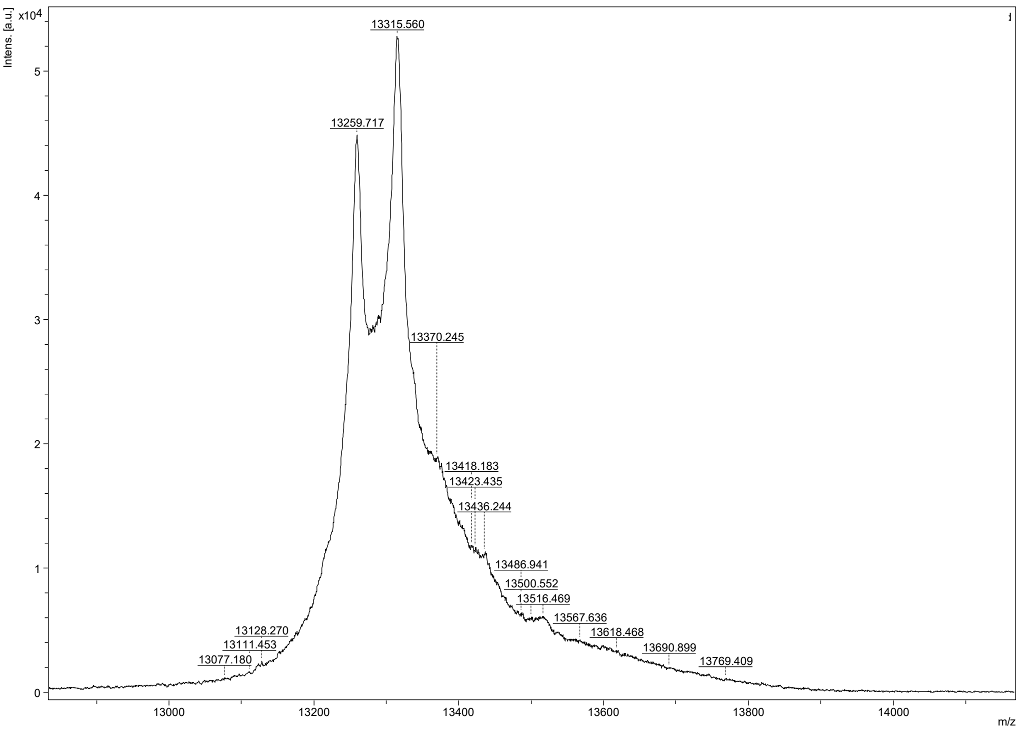

Figure S5. Mass spectroscopy analysis of enamelin. A. MALDI-TOF MS spectrum obtained after analysis of the intact protein. The expected theoretical molecular mass is 13 261 Da. Suggested assignment of the experimental m/z peaks: 13 259: 1+ monomer, 13 315: 1+ monomer with modifications such as added oxygens and sodium, 6 658: 2+ monomer, 26 574: 1+ dimer, 8 858: 3+ dimer. **B**. Zoom on the 13 kDa peak.

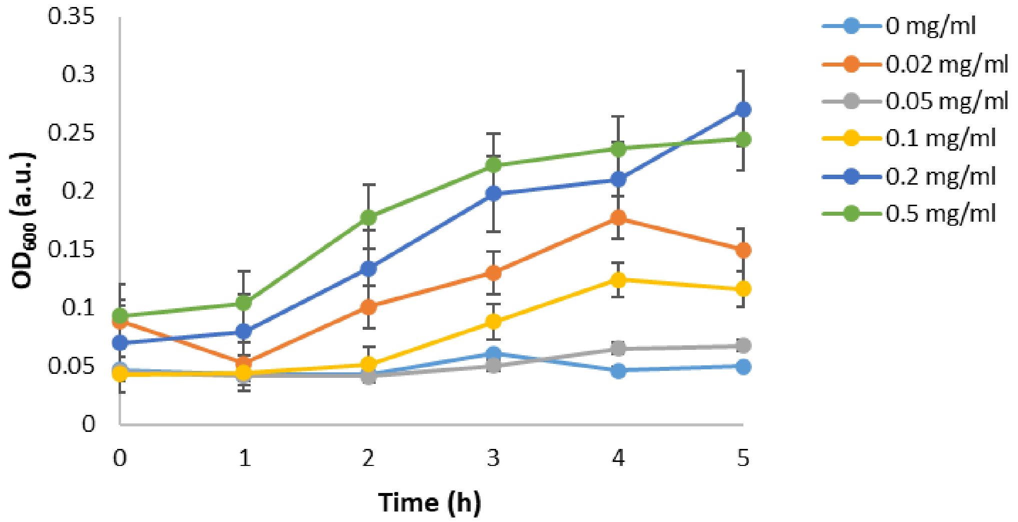

Figure S6. Mineralization experiment at different enamelin concentrations. The curves show turbidity over time as a measure for calcium phosphate precipitation. The mineralization buffer containing enamelin at various concentrations (0.02-0.5 mg/ml) is compared to buffer alone (0 mg/ml). Three separate experiments were carried out, and the values plotted are the mean values and the error bars indicate the standard error of the mean.

Table S1. MS-MS mass values after peptide fragmentation (amino acid 1-20). Fragment ions identified experimentally are highlighted in red. The following search settings were used for the MS-MS database search: trypsin as protease, one allowed missed cleavage site, 50 ppm MS accuracy for peptides and 0.5 Da MS-MS accuracy. Corresponding mass spectrum and fragmentation pattern are given in Figure S3.

Monoisotopic mass of neutral peptide Mr(calc): 2536.1315
Ions Score: 147 **Expect:** 2e-15
Matches : 76/323 fragment ions using 72 most intense peaks

#	Immon.	a	a*	a^0	b	b*	b^0	d	d'	Seq.	v	w	w'	y	y*	y^0	#
1	104.0528	104.0528			132.0478			44.0495		M							20
2	110.0713	241.1118			269.1067					H	2324.0452			2406.0983	2389.0718	2388.0878	19
3	110.0713	378.1707			406.1656					H	2186.9863			2269.0394	2252.0129	2251.0289	18
4	110.0713	515.2296			543.2245					H	2049.9274			2131.9805	2114.9540	2113.9699	17
5	110.0713	652.2885			680.2834					H	1912.8685			1994.9216	1977.8950	1976.9110	16
6	110.0713	789.3474			817.3423					H	1775.8096			1857.8627	1840.8361	1839.8521	15
7	110.0713	926.4063			954.4012					H	1638.7507			1720.8038	1703.7772	1702.7932	14
8	88.0393	1041.4333		1023.4227	1069.4282		1051.4176	997.4434		D	1523.7237	1522.7285		1583.7449	1566.7183	1565.7343	13
9	88.0393	1156.4602		1138.4496	1184.4551		1166.4446	1112.4704		D	1408.6968	1407.7015		1468.7179	1451.6914	1450.7074	12
10	88.0393	1271.4871		1253.4766	1299.4821		1281.4715	1227.4973		D	1293.6698	1292.6746		1353.6910	1336.6644	1335.6804	11
11	88.0393	1386.5141		1368.5035	1414.5090		1396.4984	1342.5243		D	1178.6429	1177.6477		1238.6640	1221.6375	1220.6535	10
12	101.1073	1514.6091	1497.5825	1496.5985	1542.6040	1525.5774	1524.5934	1457.5512		K	1050.5479	1049.5527		1123.6371	1106.6105		9
13	44.0495	1585.6462	1568.6196	1567.6356	1613.6411	1596.6145	1595.6305			A	979.5108			995.5421	978.5156		8
14	70.0651	1682.6989	1665.6724	1664.6884	1710.6938	1693.6673	1692.6833	1656.6833		P	882.4581	881.4628		924.5050	907.4785		7
15	159.0917	1868.7782	1851.7517	1850.7677	1896.7732	1879.7466	1878.7626			W	696.3787			827.4522	810.4257		6
16	101.0709	1996.8368	1979.8103	1978.8263	2024.8317	2007.8052	2006.8212	1939.8154		Q	568.3202	567.3249		641.3729	624.3464		5
17	86.0964	2109.9209	2092.8943	2091.9103	2137.9158	2120.8893	2119.9052	2081.8896	2095.9052	I	455.2361	468.2565	482.2722	513.3144	496.2878		4
18	70.0651	2206.9737	2189.9471	2188.9631	2234.9686	2217.9420	2216.9580	2180.9580		P	358.1833	357.1881		400.2303	383.2037		3
19	101.0709	2335.0322	2318.0057	2317.0217	2363.0271	2346.0006	2345.0166	2278.0108		Q	230.1248	229.1295		303.1775	286.1510		2
20	129.1135									R	74.0237	73.0284		175.1190	158.0924		1

Seq	ya	yb	Seq	ya	yb	Seq	ya	yb
HH	247.1302	275.1251	HHH	384.1891	412.1840	HHHH	521.2480	549.2429
HHHHH	658.3069	686.3018	HHHHD	636.2750	664.2699	HHHD	499.2160	527.2110
HHHDD	614.2430	642.2379	HHD	362.1571	390.1520	HHDD	477.1841	505.1790
HHDDD	592.2110	620.2059	HD	225.0982	253.0931	HDD	340.1252	368.1201
HDDD	455.1521	483.1470	HDDDD	570.1790	598.1740	HDDDDK	698.2740	
DD	203.0662	231.0612	DDD	318.0932	346.0881	DDDD	433.1201	461.1150
DDDDK	561.2151	589.2100	DDDDKA	632.2522	660.2471	DDDK	446.1882	474.1831
DDDKA	517.2253	545.2202	DDDKAP	614.2780	642.2729	DDK	331.1612	359.1561
DDKA	402.1983	430.1932	DDKAP	499.2511	527.2460	DDKAPW	685.3304	
DK	216.1343	244.1292	DKA	287.1714	315.1663	DKAP	384.2241	412.2191
DKAPW	570.3035	598.2984	DKAPWQ	698.3620		KA	172.1444	200.1394
KAP	269.1972	297.1921	KAPW	455.2765	483.2714	KAPWQ	583.3351	611.3300
KAPWQI	696.4192		AP	141.1022	169.0972	APW	327.1816	355.1765
APWQ	455.2401	483.2350	APWQI	568.3242	596.3191	APWQIP	665.3770	693.3719
PW	256.1444	284.1394	PWQ	384.2030	412.1979	PWQI	497.2871	525.2820
PWQIP	594.3398	622.3348	WQ	287.1503	315.1452	WQI	400.2343	428.2292
WQIP	497.2871	525.2820	WQIPQ	625.3457	653.3406	QI	214.1550	242.1499
QIP	311.2078	339.2027	QIPQ	439.2663	467.2613	IP	183.1492	211.1441
IPQ	311.2078	339.2027	PQ	198.1237	226.1186			
