# Supplementary material for: Recombinant human enamelin produced in Escherichia coli promotes mineralization in vitro
Source: BMC Biotechnol. 2024 Jul 9;24:48. doi: 10.1186/s12896-024-00875-0 (PMC11234762; doi:10.1186/s12896-024-00875-0)
Supplement: Supplementary file 2 — Supplementary Material 2 [file 12896_2024_875_MOESM2_ESM.pdf]

Recombinant human enamelin produced in *Escherichia coli* promotes mineralization *in vitro*.

Monalissa Halablab[1], Lovisa Wallman[1] and Johan Bonde[1*]

Affiliations:

[1]Division of Pure and Applied Biochemistry, Lund University, Lund, SE-221 00, Sweden

[*]Corresponding author email: johan.bonde@tbiokem.lth.se (J.B.)

SUPPLEMENTARY FILE

Uncropped SDS-PAGES

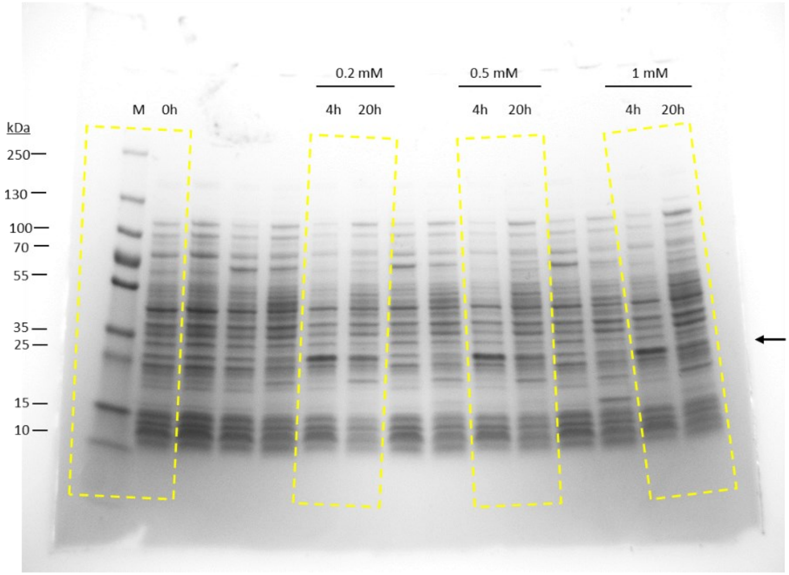

Supplementary Figure I. The parts of the SDS-PAGE gel that were cropped and shown in Figure 1 are indicated with yellow dotted box. Figure legend as in Figure 1.

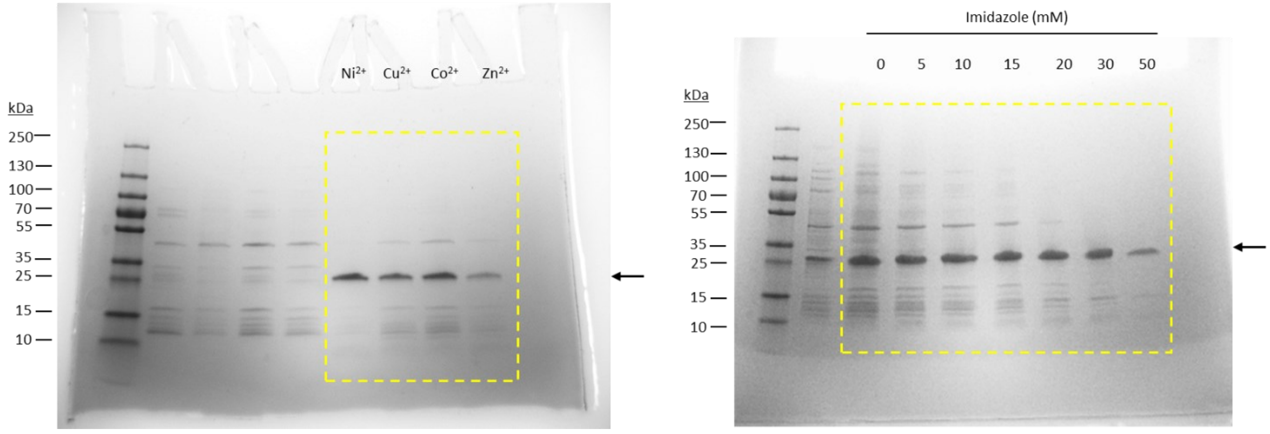

Supplementary Figure II. The parts of the SDS-PAGE gel that were cropped and shown in Figure 2A and Figure 2B are indicated with yellow dotted box. Figure legend as in Figure 2A and Figure 2B.

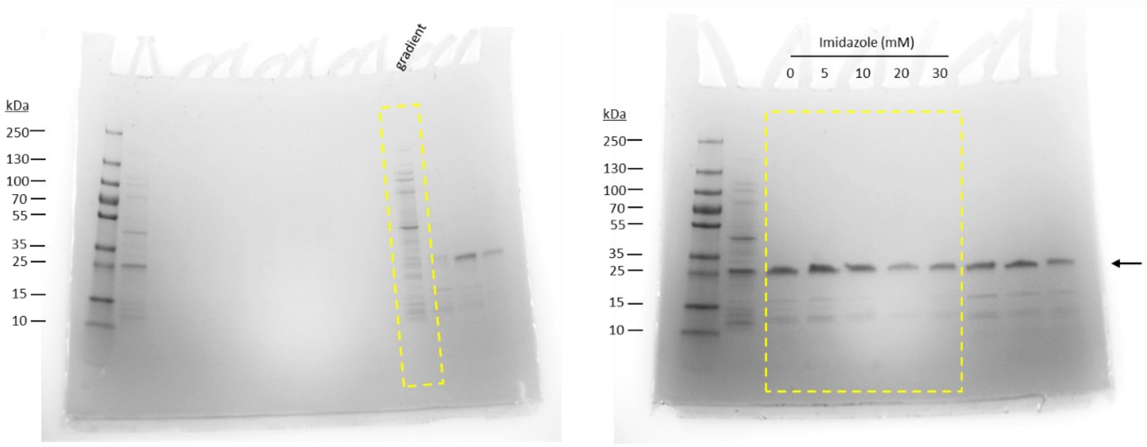

Supplementary Figure III. The parts of the SDS-PAGE gel that were cropped and shown in Figure 3B and Figure 3C are indicated with yellow dotted box. Figure legend as in Figure 3B and Figure 3C.

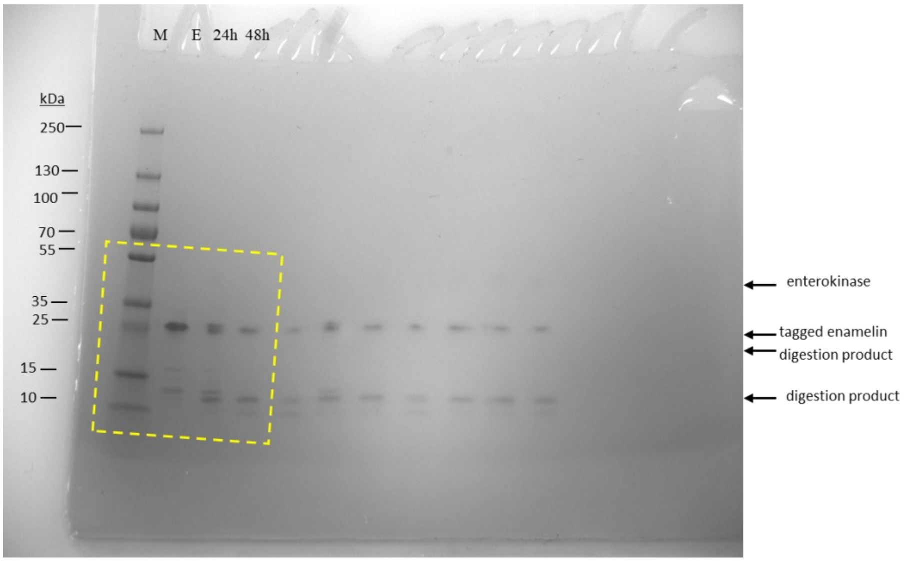

Supplementary Figure IV. The parts of the SDS-PAGE gel that were cropped and shown in Figure 4 are indicated with yellow dotted box. Figure legend as in Figure 4.

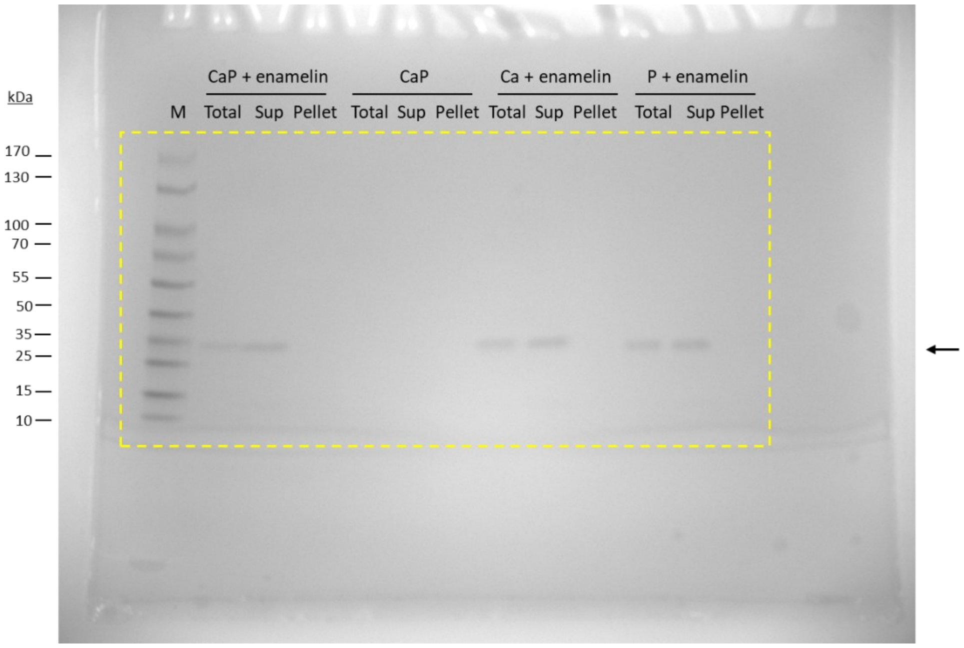

Supplementary Figure V. The parts of the SDS-PAGE gel that were cropped and shown in Figure 5B are indicated with yellow dotted box. Figure legend as in Figure 5B.
